# Sulfasalazine promotes ferroptosis through AKT-ERK1/2 and P53-SLC7A11 in rheumatoid arthritis

**DOI:** 10.1007/s10787-024-01439-6

**Published:** 2024-02-26

**Authors:** Chenyu Zhao, Yunyuan Yu, Guangrong Yin, Chao Xu, Jiahao Wang, Liangliang Wang, Gongyin Zhao, Su Ni, Haoxing Zhang, Baojun Zhou, Yuji Wang

**Affiliations:** 1 Department of Orthopedics, The Affiliated Changzhou Second People’s Hospital of Nanjing Medical University, Changzhou Medical Center, Nanjing Medical University, 29 Xinglong Alley, Changzhou, 213003 China; 2 Nanjing Medical University, 101 Longmian Avenue, Jiangning District, Nanjing, 210039 China; 3 Graduate School of Dalian Medical University, 9 West Section, Shunnan Road, Dalian, 116044 China; 4 Truma Central, The Affiliated Changzhou Second People’s Hospital of Nanjing Medical University, 29 Xinglong Alley, Changzhou, 213003 China; 5 Department of Orthopedics, Affiliated Sport Hospital of CDSU (Chengdu Sport University), 251 Wuhouci Street, Chengdu, 610041 China; 6 Department of Orthopedic Surgery and Biochemistry and Molecular Biology, Mayo Clinic, Rochester, MN USA; 7 Medical Research Center, The Affiliated Changzhou Second People’s Hospital of Nanjing Medical University, 29 Xinglong Alley, Changzhou, 213003 China; 8 Guangdong Provincial Key Laboratory of Genome Stability and Disease Prevention, College of Life Sciences and Oceanography, Shenzhen University, Shenzhen, 518055 People’s Republic of China; 9 Department of Orthopedics, The Third Affiliated Hospital of Gansu University of Chinese Medicine, 222 Silong Road, Baiyin, 730900 China

**Keywords:** Ferroptosis, Rheumatoid arthritis, Sulfasalazine, Fibroblast-like synoviocyte

## Abstract

**Objective:**

Ferroptosis has been reported to play a role in rheumatoid arthritis (RA). Sulfasalazine, a common clinical treatment for ankylosing spondylitis, also exerts pathological influence on the progression of rheumatoid arthritis including the induced ferroptosis of fibroblast-like synoviocytes (FLSs), which result in the perturbated downstream signaling and the development of RA. The aim of this study was to investigate the underlying mechanism so as to provide novel insight for the treatment of RA.

**Methods:**

CCK-8 and Western blotting were used to assess the effect of sulfasalazine on FLSs. A collagen-induced arthritis mouse model was constructed by the injection of collagen and Freund’s adjuvant, and then, mice were treated with sulfasalazine from day 21 after modeling. The synovium was extracted and ferroptosis was assessed by Western blotting and immunofluorescence staining.

**Results:**

The results revealed that sulfasalazine promotes ferroptosis. Compared with the control group, the expression levels of ferroptosis-related proteins such as glutathione peroxidase 4, ferritin heavy chain 1, and solute carrier family 7, member 11 (SLC7A11) were lower in the experimental group. Furthermore, deferoxamine inhibited ferroptosis induced by sulfasalazine. Sulfasalazine-promoted ferroptosis was related to a decrease in ERK1/2 and the increase of P53.

**Conclusions:**

Sulfasalazine promoted ferroptosis of FLSs in rheumatoid arthritis, and the PI3K-AKT-ERK1/2 pathway and P53-SLC7A11 pathway play an important role in this process.

## Introduction

Rheumatoid arthritis (RA) is a chronic inflammatory joint disease, which could cause deformity of the joints and physical disability (Aira et al. 2016). Moreover, clinical data indicated that RA is more predominant among women, particularly within the age group of 20–40. The early symptoms of RA usually occur in small joints, such as the hands and feet (Guo et al. 2018). According to an estimation from The United States Centers for Disease Control and Prevention, the global incidence of RA is approximately 0.5–1% (Shaaban et al. 2022). RA reduces the quality of life and shortens the lives of those affected (Shaw et al. 2018). As RA progresses, the synovium of the joint transforms into proliferative invasive tissue, accompanied by an increase in the secretion of inflammatory mediators. Consequently, the cartilage degenerates, which ultimately leads to bone destruction (Montero-Melendez et al. 2020).

Fibroblast-like synoviocytes (FLSs) play important roles in inflammatory mediator production, which promotes the deterioration of RA (Guma et al. 2015). However, the underlying mechanism is not clear.

Ferroptosis is a special type of programmed cell death different from other forms of cell death such as autophagy, pyroptosis, and apoptosis (Dixon et al. 2012). Ferroptosis is promoted by iron-dependent lipid peroxidation, principally manifesting as an increase in mitochondrial membrane density and a reduction in or disappearance of mitochondrial cristae (Qu et al. 2021; Zhang et al. 2022). During ferroptosis, mitochondria will decrease in size and the mitochondrial outer membrane will rupture (Zhou et al. 2022). Ferroptosis could be induced by iron metabolism disorder, lipid peroxidation accumulation, deficiency of glutathione (GSH) and inactivation of the antioxidant enzyme glutathione peroxidase 4 (GPX4) (Chen et al. 2019; Yuan et al. 2022). Ferroptosis comprises two major biochemical processes: iron accumulation and lipid peroxidation. Excessive iron accumulation acts as a catalyst for redox reactions, leading to oxidative stress and cytotoxicity. Iron ions exist in two forms in cells, Fe^3+^ and Fe^2+^. Fe^2+^ activates cellular reactive oxygen species (ROS) through the Fenton reaction, leading to the accumulation of lipid peroxides, which further promotes lipid peroxidation (Canli et al. 2016; Hirayama et al. 2017). Lipid peroxidation affects the metabolism of unsaturated fatty acids, especially poly unsaturated fatty acids in cell membranes, which increase the level of aldehydes (e.g., malondialdehyde (MDA) and 4-hydroxynonenal (4HNE) during ferroptosis process (Llabani et al. 2019; Han et al. 2020).

Proinflammatory factors such as interleukin-1β (IL-1β), IL-6, IL-7, IL-8 and tumor necrosis factor-α (TNF-α) are involved in RA pathogenesis. The modulation of TNF-α, IL-1β, and IL-6 expression has been widely used to treat RA (Akram et al. 2021). Li et al. found that ferroptosis plays a crucial role in autoimmune and inflammatory diseases (Li et al. 2021). In addition, Ling et al. found that ferroptosis exerts an important influence on maintaining the balance between synovial proliferation and death (Ling et al. 2022). These studies indicated that ferroptosis might participate in the pathological process of RA.

Sulfasalazine is chemically composed of 5-aminosalicylic acid (5-ASA) and sulfapyridine (SP) both of which share the linkage with azo bonds. Although used as an anti-inflammatory drug for inflammatory arthritis and inflammatory bowel disease, SP may also be able to be used to treat RA (Zheng et al. 2020). Nevertheless, the molecular mechanism of sulfasalazine in the treatment of RA remains unclear (Jansen et al. 2015). In this study, we investigated the effect and underlying mechanism of sulfasalazine on RA FLS ferroptosis to provide novel insight and strategies for the treatment of RA.

## Materials and methods

### Reagents

Sulfasalazine was purchased from Sigma (CAS:599-79-1). DFO, LM-22B, and pifithrin-α were purchased from MCE. BODIPY 581/591 C11 (USA, D3861) was purchased from Thermo Fisher. All ELISA kits were purchased from MultiSciences Biotech, Co., Ltd. Primary and secondary antibodies were provided by Proteintech (Wuhan, China) and ABclonal (Wuhan, China).

### Isolation and culture of human fibroblast synoviocytes

RA samples were washed with phosphate-buffered saline (PBS) to remove adipose tissue from the synovium. All the samples were collected from patients in which informed consent was obtained for experimentation with human subjects. The process strictly followed the Code of Ethics of the World Medical Association (Declaration of Helsinki). RA tissue was digested for 12 h in serum-free Dulbecco’s modified Eagle’s medium (DMEM) containing 1 mg/ml collagenase type I. Finally, the cells were harvested after filtration with the 70-μmol filter. The extracted cells were cultured in DMEM containing 10% fetal bovine serum (FBS), 100 U penicillin at 37 °C in 5% carbon dioxide. Adherent cells were passaged at a ratio of 1:2, and cells from passages 2–4 were used in our experiment.

### Scratch test

Lines were drawn on the back of six-well plate with marker pen and 5 × 10^5^ cells were added to each well. After the cells were confluent, scratches were made with the pipette tip perpendicular to the horizontal line. Low-serum medium containing the treatment indicated foreach group was added to each well. Cells were cultured in an incubator with 5% CO_2_ at 37 °C and then observed under microscope.

### Cell viability assay

Cell viability was assessed by Cell Counting KIT-8 (CCK-8). FLSs were cultured in 96-well plates at a density of 6 × 10^3^ cells per well. After 12 h, FLSs were pretreated with TNF-α for 4 h, and then the culture medium was discarded. After treatment with different reagents, the medium was discarded, then 100 μl of culture medium and 10 μl of CCK-8 solution were added to each well. After further incubation for 2 h, the absorbance was measured at 450 nm with a microplate reader (Elx808™ Bio-Tek Instruments, Winooski, VT, USA).

### EDU incorporation assay

FLSs were seeded in 24-well cell culture plates (2 × 10^4^ cells per well). FLSs pretreated with TNF-α for 4 h, and then, the culture medium was discarded. After treatment with different drugs, 5-ethynyl-2′-deoxyuridine (EDU) working solution (Liquid A) was added. Cells were fixed with 4% paraformaldehyde for 30 min, followed by the addition of 2 mg/ml glycine per well for 5 min and 0.5% Triton X-100 for 20 min. An EDU test was carried out according to the EDU instructions. Finally, DNA staining was performed and the cells were observed under a microscope.

### Transwell assay

The migration and invasion abilities of FLSs were assessed with 8-μm pore transwell chambers. Two hundred microliters of serum-free DMEM containing 2 × 10^5^ FLSs/ml was added to the upper chamber, and 600 μl complete medium was added to the lower chamber. After drug treatment, the cells were incubated for 48 h and then fixed with 4% paraformaldehyde for 1 h. The cells in the upper chamber were removed with cotton swabs and imaged by optical microscopy (Olympus, China).

### Colony formation assay

After drug treatment, the cells were washed with PBS and harvested with 0.05% trypsin/EDTA solution. Next, 200 cells from each group were seeded into a six-well plate. After a 2-week incubation, colonies were fixed with 100% methanol for 30 min. Subsequently, colonies were stained with crystal violet solution for 30 min and dried overnight before evaluation.

### ELISA analysis

Cells were incubated for 48 h after sulfasalazine treatment. The supernatant was collected, and after centrifugation, a matrix metalloproteinases-3 (MMP-3) ELISA kit, MMP-13 ELISA kit, IL-1β ELISA kit, IL-6 ELISA kit, and TNF-α ELISA kit were used according to the manufacturer’s instructions.

### ROS detection

FLSs were evenly distributed in 24-well plates with a density of 3–4 × 10^4^ cells per well. After incubation for 24 h at 37 °C in a cell incubator, cells were washed three times with PBS and then treated with 5 µM C11 BODIPY for 30 min in the dark in the cell incubator. Finally, the specimens were observed under a fluorescence microscope (Nikon Eclipse Ti, Japan).

### Western blot analysis

The FLSs were distributed on a six-well plate with a density of 4–5 × 10^5^ cells per well. The FLSs were lysed with 150 μl radioimmunoprecipitation assay buffer (RIPA) (Beyotime, Shanghai, China) containing 1% phenylmethanesulfonyl fluoride (PMSF) and 1% cocktail for 20 min on ice. Then they were treated with an ultrasonic crushing apparatus and boiled for 10 min. Protein samples were electrophoresed on 12% SDS-PAGE gels and transferred to polyvinylidene difluoride (PVDF) membranes. Then, membranes were blocked with 5% skim milk for 1–1.5 h at room temperature and incubated overnight with primary antibodies. Next, the membranes were incubated with secondary antibodies for 1–1.5 h at room temperature. Protein bands were detected by using a high-sensitivity ECL chemiluminescence kit (New Cell & Molecular Biotech Co. Ltd), and the relative expression levels of proteins were quantified by ImageJ software.

### Immunofluorescence staining

FLSs were seeded in 24-well plates containing slides. The cells were fixed with 4% paraformaldehyde for 30 min at room temperature. After permeation with 0.5% Triton X-100 for 30 min at room temperature, 5% albumin from bovine serum (BSA) was used for blocking. The cells were incubated with primary antibody diluted with 5% BSA (1:100) overnight at 4 °C. After treatment, the cells were washed and incubated with secondary antibody (1:100) for 1 h at room temperature in the dark. The cells were sealed with a fluorescence quencher containing 4’,6-diamidino-2-phenylindole (DAPI) and observed under a fluorescence microscope.

### Animal experiment

CIA model development

The process was strictly followed the National Research Council’s Guide for the Care and Use of Laboratory Animals. The mice were injected with collagen and complete Freund’s adjuvant 0.5 cm at the tail root. Twenty-one days after the treatment, the mice were injected with collagen and incomplete Freund’s adjuvant at a different position (enhanced immunity) 0.5 cm from the tail root.

**Groups**

Forty mice were randomly divided into four groups with ten mice in each group.

Group 1: control group.

Group 2: CIA group.

Group 3: CIA + sulfasalazine (SASP) group (50 mg/kg, three times a week).

Group 4: CIA + methotrexate (MTX) group (5 mg/kg, three times a week).

All mice were fed a high-quality diet during treatment. The mice were killed on the 56th day, the knees and paws were harvested for imaging and pathological examination.

### Immunohistochemistry

Synovial tissue was obtained from DBA/1 mice and was sectioned after being embedded in paraffin. Dewaxing, antigen retrieval, peroxidase inhibition, blocking, incubation with primary and secondary antibodies, diaminobenzidine (DAB) color development and sealing were all carried out following the instructions.

### Hematoxylin–eosin staining and safranin-fast green staining

A hematoxylin–eosin/HE staining kit (Solarbio) or Modified Saffron-O and Fast Green Stain Kit (Solarbio) was used for hematoxylin–eosin staining or safranin-fast green staining. Paraffin section dewaxing, hematoxylin–eosin staining, safranin-fast green staining, dehydration, sealing, microscopic observation, all the above operations are carried out according to the instructions.

### Mouse arthritis index score

The redness and swelling of the paws of each mouse in each group were scored, with the highest score being 16 for each mouse. The scoring criteria were as follows: normal: 0; slight swelling of ankle joint: 1; slight swelling of ankle to metatarsal and metacarpal joints: 2; moderate swelling of ankle to metatarsal and metacarpal joints: 3; severe swelling of ankle to metatarsal and metacarpal joints: 4.

### Statistical analysis

The data in this study are presented as the mean ± SD of at least three independent experiments. Differences between the two groups were compared with Student’s *t* test, and one-way ANOVA was used for comparisons of more than two groups. All reported *p* values were two-tailed, and *p* values less than 0.05 were considered statistically significant.

## Results

### Ferroptosis is inhibited in RA FLSs

**Fig. 1:**
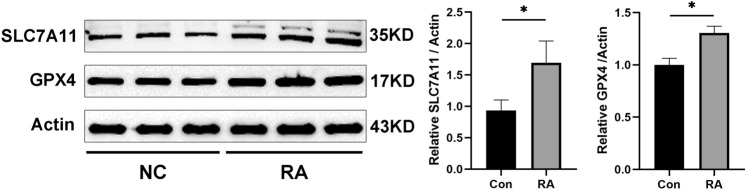
FLS ferroptosis in rheumatoid arthritis. Three pairs of control/RA synovium were ground in liquid nitrogen, and Western blotting was used to detect and quantification the expression of the ferroptosis-related proteins GPX4 and SLC7A11. Student’s *t* test was used to analyze the experimental data (*p < 0.05, **p < 0.01, ***p < 0.001, ^#^*p* < 0.0001)

We measured the protein levels of GPX4 and SLC7A11, the two hallmarks of ferroptosis in paired synovial specimens from RA and trauma patients. The results showed that the total levels of GPX4 and SLC7A11 in RA FLSs were significantly higher than those in control group (Fig. 1). The suppressed ferroptosis in FLSs from RA patients might account for the survival of FLSs as well as the resistance of RA patients to current therapy.

### Sulfasalazine inhibits FLS proliferation

**Fig. 2:**
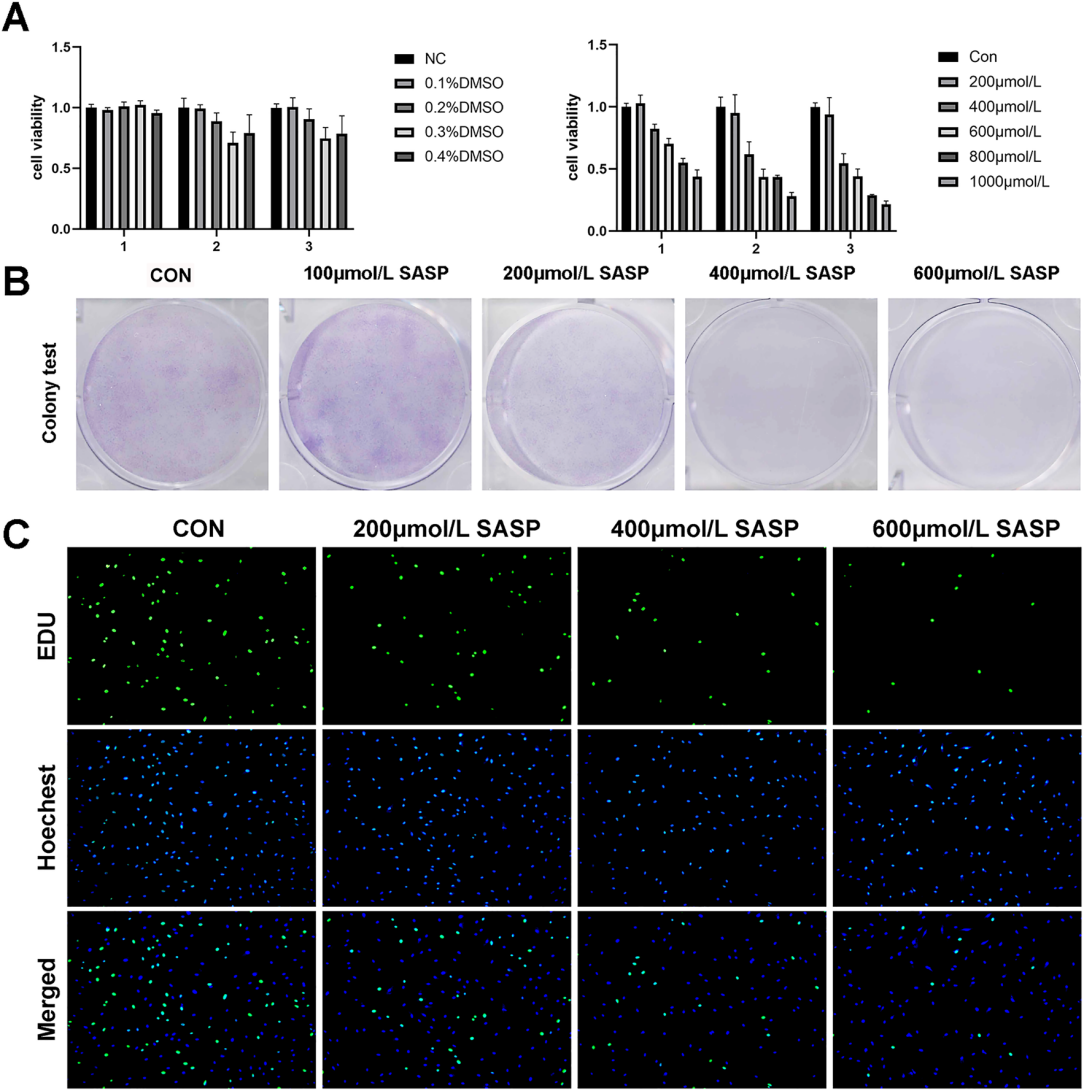
SASP can inhibit the proliferation of FLSs. FLSs were divided into five groups: NC group, 0.1% DMSO group, 0.2% DMSO group, 0.3% DMSO group and 0.4% DMSO group. After grouping, CCK-8 was used to analyze cell viability at 24, 48, and 72 h. FLSs were then divided into four groups: control group, 200 μmol/l SASP, 400 μmol/l SASP, 600 μmol/l SASP (all groups contained 0.2% DMSO solvent and were pretreated with TNF-α for 4 h). **A** Cell viability was analyzed by CCK-8 at 24, 48, and 72 h. **B** The proliferation capacity of the cells was analyzed by cell colony assay after 14 days of treatment. **C** Original magnification, ×100. EDU experiment was conducted after 48 h of treatment. The* green spots* represent proliferating cells. Student’s *t* test was used to analyze the experimental data (*p < 0.05, **p < 0.01, ***p < 0.001, ^#^*p* < 0.0001)

We analyzed the effects of sulfasalazine on the proliferation of RA FLSs. Different concentrations of sulfasalazine were used to treat FLSs for 2 days before TNF-α treatment. The results of CCK-8 assay and EDU incorporation analysis showed the decreased cell viability upon sulfasalazine treatment in a dose-dependent manner (Fig. 2A, C). Additionally, according to the results of colony-formation experiments, the number of cell colonies decreased significantly, increasing with drug concentration (Fig. 2B). All these data indicate sulfasalazine can inhibit the proliferation of FLSs.

### Sulfasalazine inhibits FLS migration and invasion

**Fig. 3:**
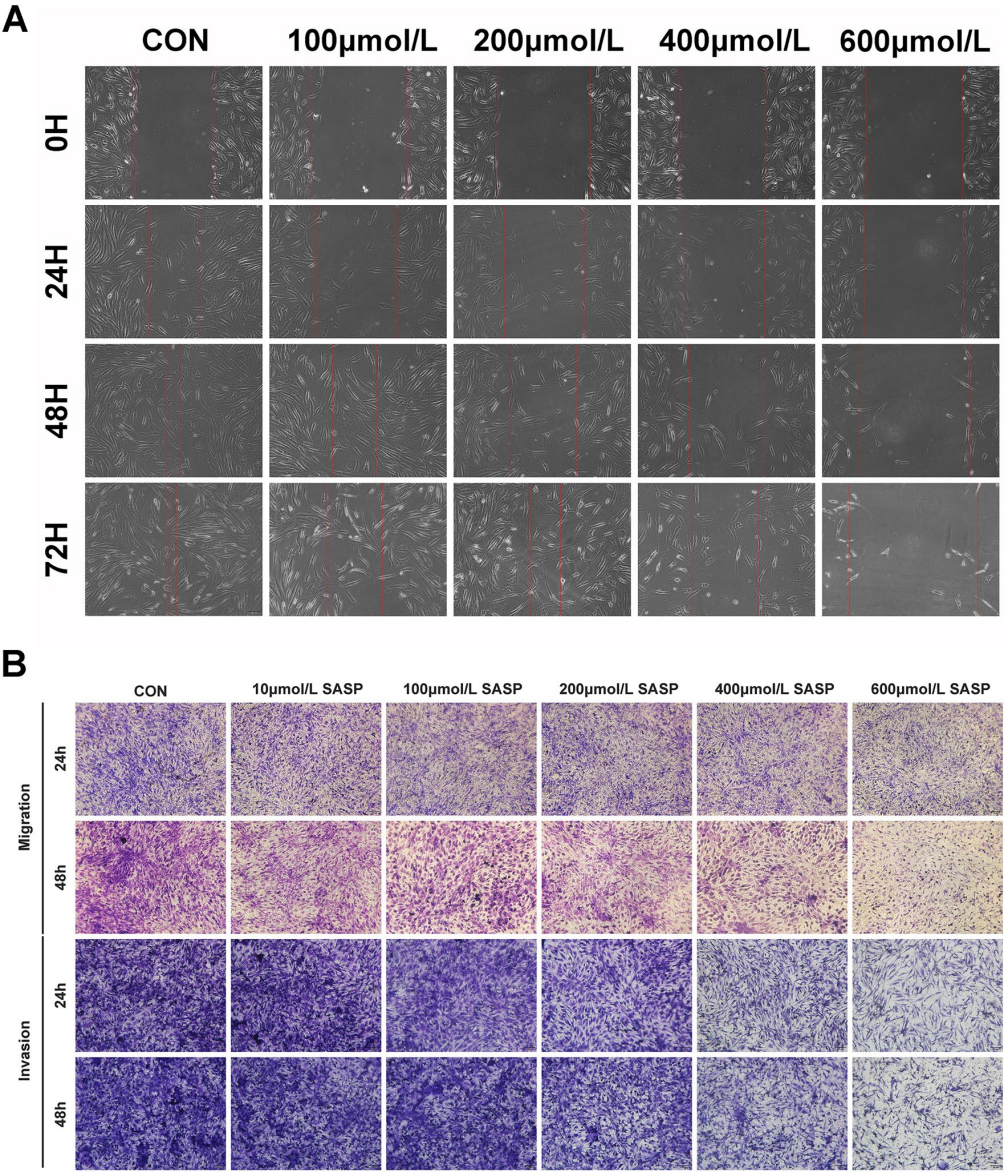
SASP can inhibit FLSs migration and invasion. FLSs were divided into six groups: control group, 10 μmol/l SASP, 100 μmol/l SASP, 200 μmol/l SASP, 400 μmol/l SASP, 600 μmol/l SASP (all groups contained 0.2% DMSO solvent and were pretreated with TNF-α for 4 h). **A** Cell proliferation and migration were analyzed by scratch assay at 0,  h, 48, and 72 h. **B** Cell migration and invasion ability were analyzed by transwell assay at 24 and 48 h

Next, the scratch test and transwell test were employed to evaluate the effects of sulfasalazine treatment on the cellular migration and invasion of FLSs. As shown in Fig. 3, increase sulfasalazine concentration result in significantly decreased the number of synovial membrane cell metastasis and invasion (Fig. 3A–C).

### Sulfasalazine inhibits proinflammatory markers in FLSs

**Fig. 4:**
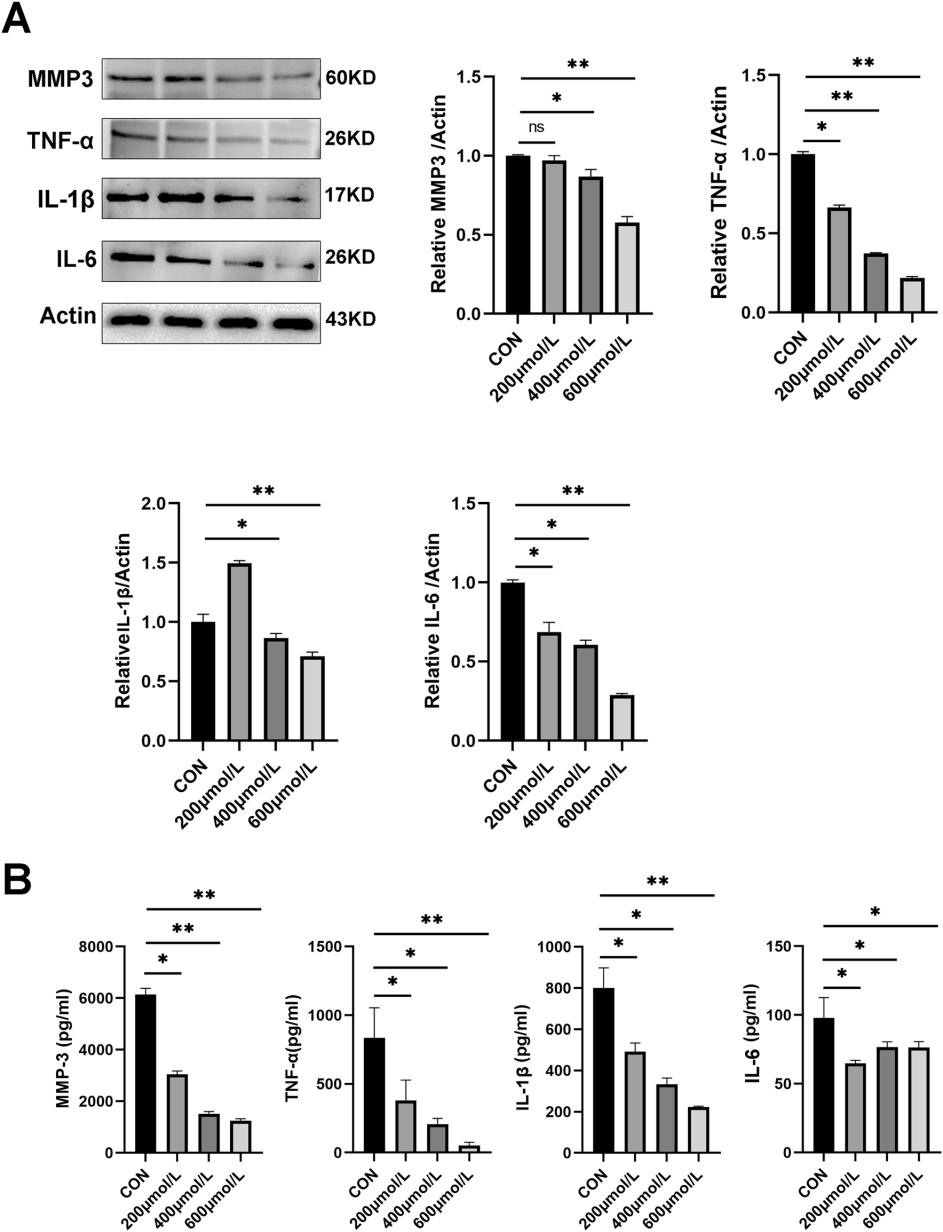
SASP can inhibit the production of inflammatory cytokines in FLSs. FLSs were divided into four groups: control group, 200 μmol/l SASP, 400 μmol/l SASP, 600 μmol/l SASP (all groups contained 0.2% DMSO solvent and were pretreated with TNF-α for 4 h). **A** After 48 h of treatment, the levels of MMP3, MMP13, IL-1β, and IL-6 were assessed by Western blot. **B** The levels of MMP3, MMP13, IL-1β, and IL-6 were detected by ELISA. Student’s *t* test was used to analyze the experimental data (*p < 0.05, **p < 0.01, ***p < 0.001, ^#^*p* < 0.0001)

We also measured the effect of sulfasalazine treatment on the proinflammation pathway. Western blot results showed that the protein levels of inflammation-related markers including MMP3, MMP9, IL-1β, and IL-6 were gradually down-regulated after sulfasalazine treatment (Fig. 4A). Moreover, ELISA results were similar to the trends observed in Western blots (Fig. 4B). These data suggest that sulfasalazine treatment negatively regulates proinflammatory signaling in FLSs.

### Sulfasalazine promotes ferroptosis in FLSs

**Fig. 5:**
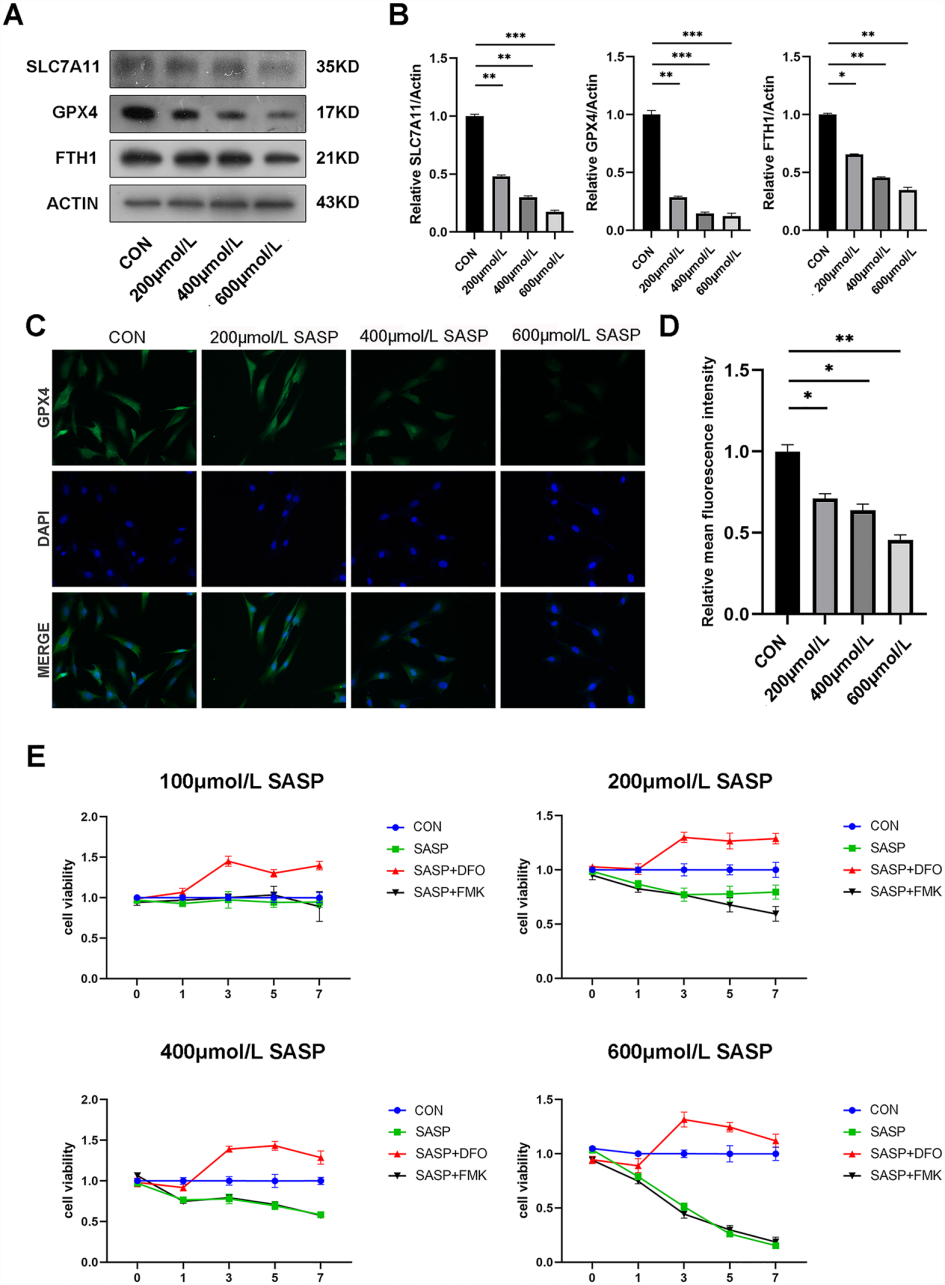

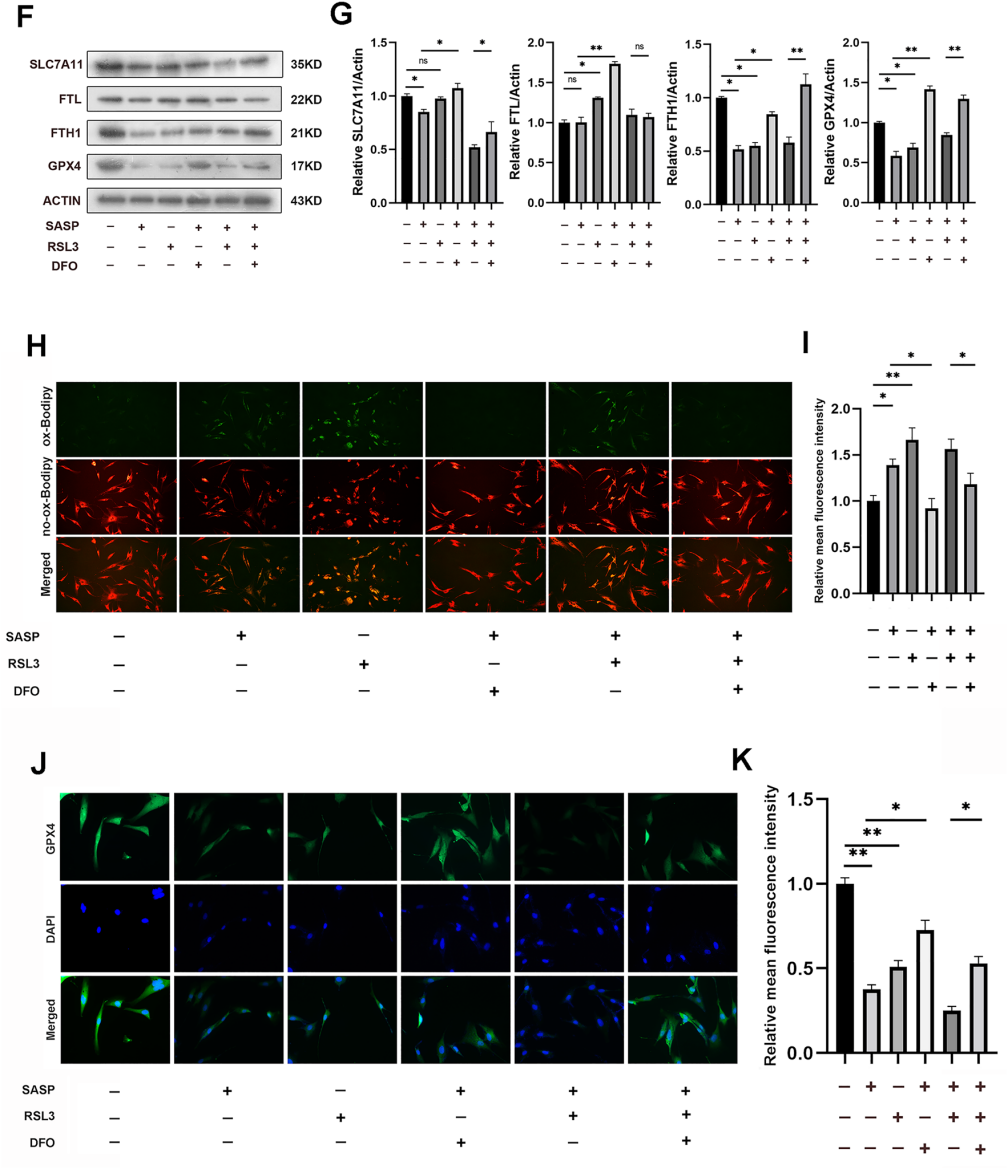
SASP promotes FLSs ferroptosis. FLSs were divided into four groups: control group, 200 μmol/l SASP, 400 μmol/l SASP, 600 μmol/l SASP (all groups contained 0.2% DMSO solvent and were pretreated with TNF-α for 4 h). **A**, **B** The levels of GPX4, FTH1, and SLC7A11 were detected and quantified by Western blot after 48 h treatment. **C**, **D** Post-original magnification, ×400. After 48 h of treatment, immunofluorescence and quantification were performed. **E** FLSs were divided into four groups: control group, 400 μmol/l SASP, 400 μmol/l SASP + DFO, 400 μmol/l SASP + FMK (all groups contained 0.2% DMSO solvent and were pretreated with TNF-α for 4 h). Cell viability was analyzed by CCK-8 on day 0, day 1, day 3, day 5, and day 7. FLSs were divided into six groups: control group, 400 μmol/l SASP, 400 μmol/l RSL3, 400 μmol/l SASP + DFO, 400 μmol/l SASP + RSL3 and 400 μmol/l SASP + RSL3 + DFO (all six groups contained 0.2% DMSO solvent and were pretreated with TNF-α for 4 h). **F**, **G** After 48 h of treatment, the levels of GPX4, FTH1, FTL, and SLC7A11 were detected and quantified by Western blot. **H**, **I** Original magnification, ×200. The intracellular accumulation of lipid peroxidation was analyzed by BODIPY-C11 assay. Each* dot* represents an individual value, and* no dot* represents a comparison. **J**, **K** Original magnification, ×400. After 48 h of treatment, immunofluorescence, and quantification were performed. Student’s* t* test was used to analyze the experimental data (*p < 0.05, **p < 0.01, ***p < 0.001, ^#^*p* < 0.0001)

Ferroptosis-related proteins (GPX4, SLC7A11, FTH1) have been reported to be negatively correlated with ferroptosis in multiple cells. In FLSs, sulfasalazine treatment decreased the expression levels of ferroptosis-related proteins (Fig. 5A, B). Immunofluorescence staining showed similar results (Fig. 5C, D). Using CCK-8 assay, we discovered that DFO (ferroptosis inhibitor) could reverse the FLSs ferroptosis induced by SASP while apoptosis inhibitor FMK had no obvious effect (Fig. 5E). Western blotting data showed that both SASP and RSL3 (ferroptosis inducer) could inhibit the expression of ferroptosis-related proteins and both could be reversed by DFO (Fig. 5F, G). Similar results were obtained by immunofluorescence staining (Fig. 5J, K). To further determine the effect of sulfasalazine on lipid peroxidation, BODIPY-C11 assay was performed and the results showed that both SASP and increased the reactive oxygen species level in FLSs, an effect that could be blocked by inhibiting ferroptosis with DFO. The above data indicated that sulfasalazine promotes FLS ferroptosis and lipid peroxidation.

### Sulfasalazine promotes ferroptosis in FLSs through PI3K-AKT-ERK1/2 and P53 pathways

**Fig. 6:**
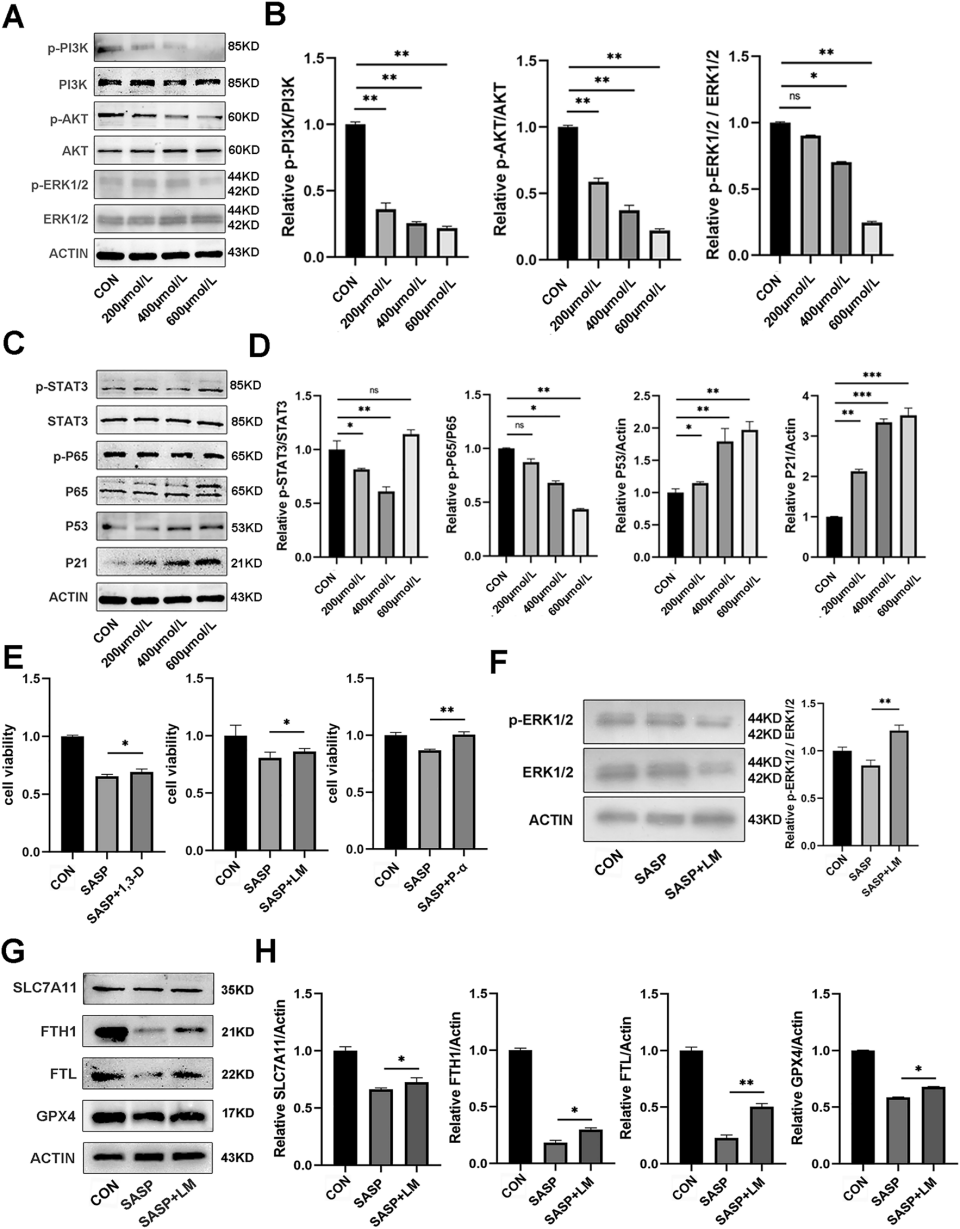

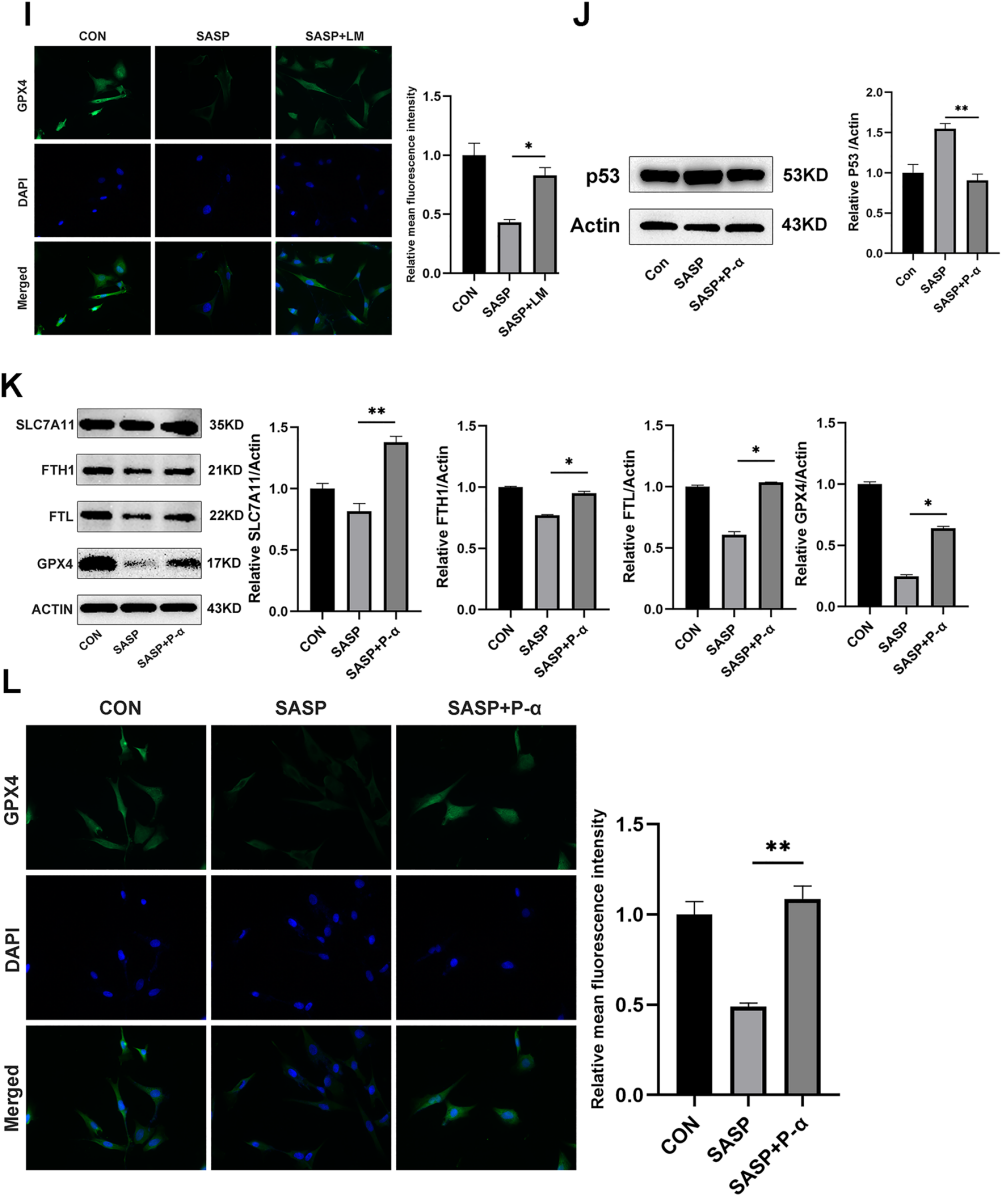
SASP promotes ferroptosis through PI3K-AKT-ERK1/2 and P53 pathways. FLSs were divided into four groups: control group, 200 μmol/l SASP, 400 μmol/l SASP, 600 μmol/l SASP (all groups contained 0.2% DMSO solvent and were pretreated with TNF-α for 4 h). **A**–**D** After 10 min of treatment, the expression levels and quantification of p-PI3K, PI3K, p-AKT, AKT, p-ERK1/2, ERK1/2, P53, P21, P65, p-P65, p-STAT3, and STAT3 were detected by Western blot. **E** Cell activity after treatment with PI3K-AKT agonist 1,3-D, PI3K-Akt-erk1/2 agonist LM-22B, P53 inhibitor P-α. **F** Expression levels and quantification of p-ERK1/2 and ERK1/2 after LM-22B treatment. **G**, **H** Expression levels and quantification of GPX4, FTH1, FTL, and SLC7A11 after LM-22B treatment. **I** Original magnification, ×400. Immunofluorescence and quantification. **J** Expression level and quantification of P53 after P-α treatment. **K** Expression levels and quantification of GPX4, FTH1, FTL, and SLC7A11 after P-α treatment. **L** Original magnification, ×400. Immunofluorescence and quantification. Student’s* t* test was used to analyze the experimental data (*p < 0.05, **p < 0.01, ***p < 0.001, ^#^*p* < 0.0001)

To elucidate the molecular mechanism involved in sulfasalazine-induced ferroptosis in FLSs, we analyzed, by Western blot analysis, the expression levels of a series of important signaling proteins including p-PI3K/PI3K, p-AKT/AKT, p-ERK1/2/ERK1/2, in FLSs treated with different concentrations of sulfasalazine for 10 min. The results showed that SASP downregulated p-PI3K/PI3K, p-AKT/AKT and p-ERK1/2/ERK1/2 expressions while up-regulate P53 and P21 expression (Fig. 6A–D). CCK-8 assay results showed that after addition of PI3K-AKT-ERK1/2 pathway activators LM-22B and 1,3-D and P53 inhibitor P-α, the decrease in cell viability resulting from SASP treatment was recovered. In particular, P53 inhibition resulted in a markedly stronger recovery phenotype than other chemical manipulations (Fig. 6E). In addition, compared with SASP treatment alone, treatment with PI3K-AKT-ERK1/2 activator LM-22B increased the p-ERK1/2 and ERK1/2 levels (Fig. 6F) and ferroptosis-related proteins (GPX4, SLC7A11, FTH1, FTL) (Fig. 6G,H). These results were also verified by immunofluorescence staining (Fig. 6I). After the addition of a P53 pathway inhibitor, the downregulation of ferroptosis-related proteins was reversed (Fig. 6J–L), and similar results were obtained by immunofluorescence staining (Fig. 6M). In summary, these data demonstrate that both PI3K-AKT-ERK1/2 and P53 pathways are important for sulfasalazine-promoted ferroptosis in FLSs.

### Ferroptosis is involved in the sulfasalazine treatment on RA in vivo

**Fig. 7:**
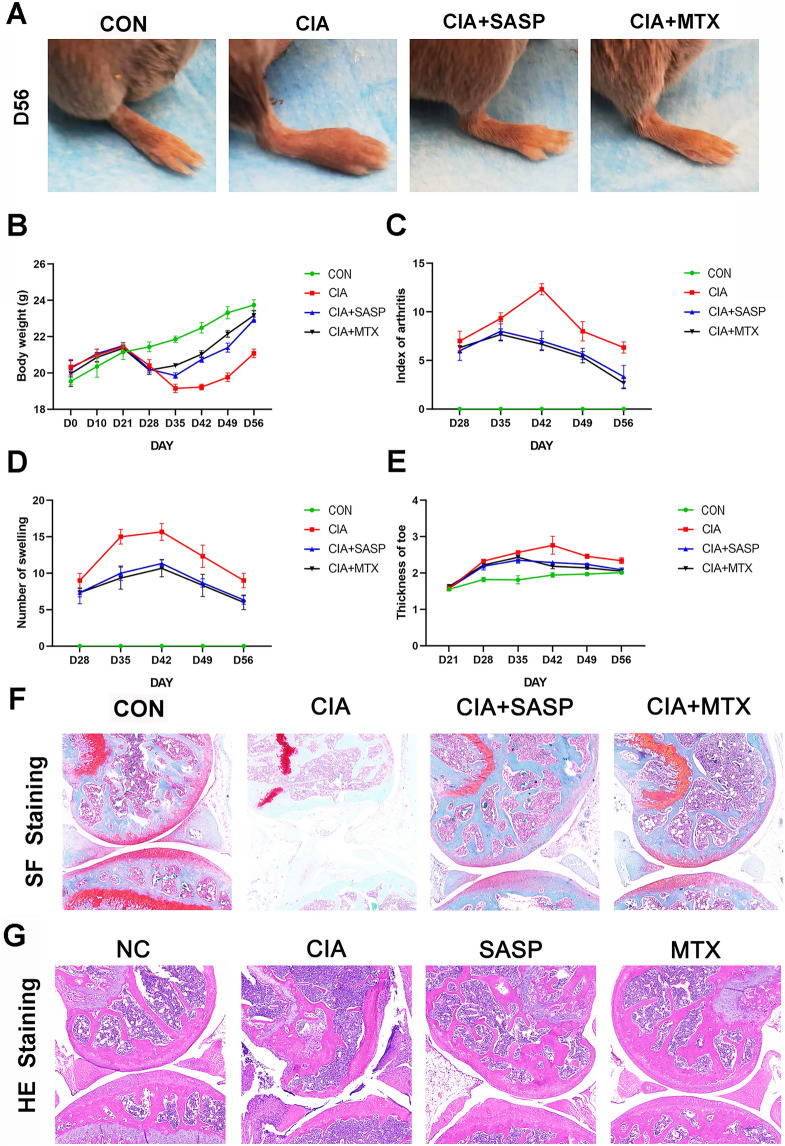

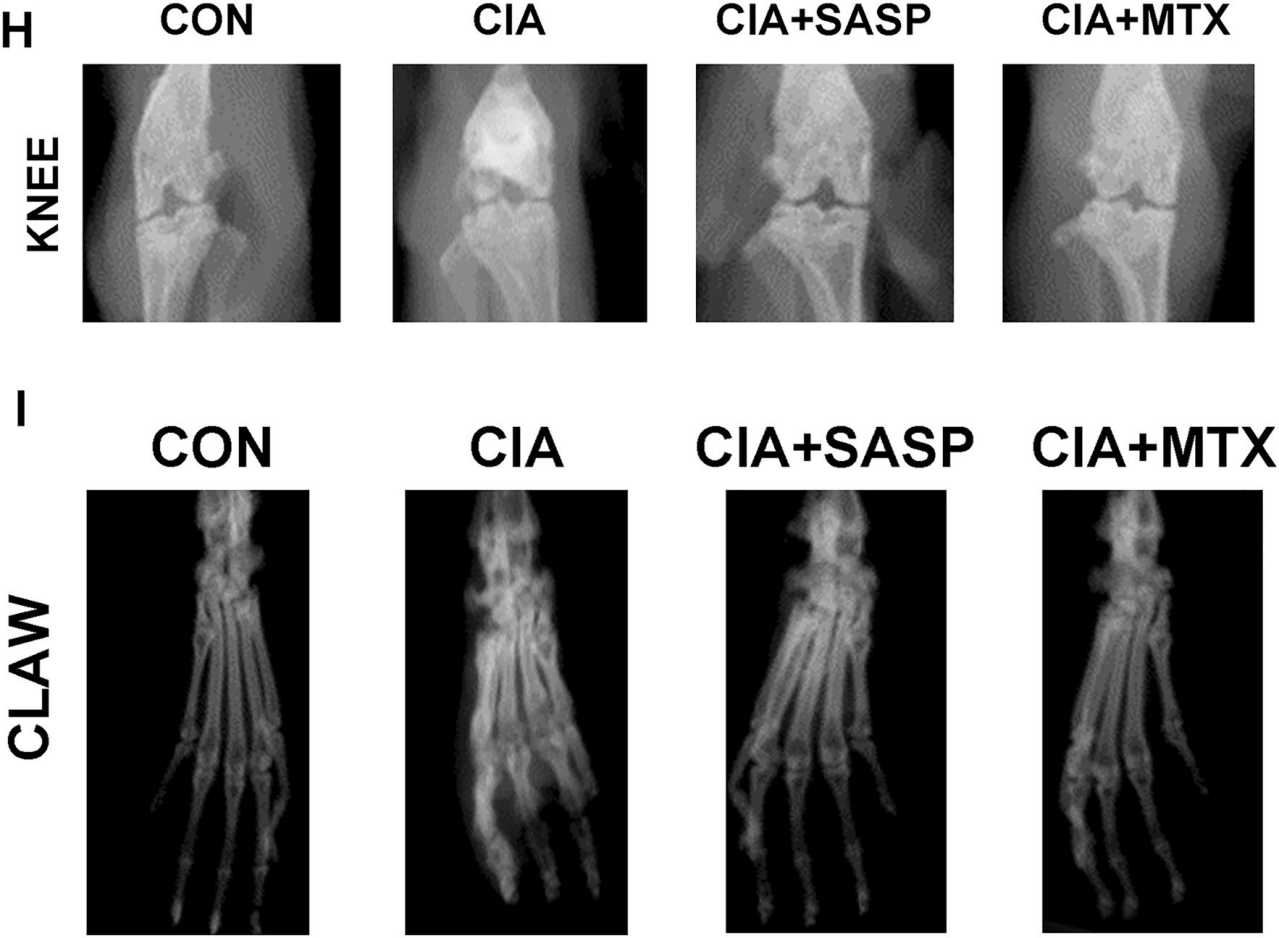
SASP treatment of RA in CIA mice. DBA/1 mice were divided into four groups: control group, CIA group, CIA + SASP group, and CIA + MTX group. **A** Paw changes in mice in the four groups at 56 days. **B**–**E** Body weight, arthritis index, swelling, and thickness of the toe of mice in four groups within 56 days. **F** Original magnification, ×100. H&E staining of mouse knee joint. **G** Original magnification, ×100. S&F staining of mouse knee joints. **H**, **I** Imaging observation of mouse knee joint and claw. Student’s *t* test was used to analyze the experimental data (*p < 0.05, **p < 0.01, ***p < 0.001, ^#^*p* < 0.0001)

CIA model mice were successfully established 5 weeks after beginning modeling. The swelling of mouse paws was more obvious than in the control group, and this phenotype could be reversed by treatment with SASP and MTX (Fig. 7A). CIA mice reached their lowest value on day 35. The arthritis score, number of swelling events, and degree of toe swelling peaked on day 42, and the rescue of these phenotypes was also observed following SASP and MTX treatment (Fig. 7B–E). To estimate the pathological impacts, H&E staining and S&F staining were conducted. The results showed that synovial hyperplasia and cartilage destruction occurred more progressively in CIA group than in the control group. The changes were reversed in SASP- and MTX-treated groups (Fig. 7F, G). Furthermore, our radiological examination results showed that more severe bone and cartilage damage of both knee joints and paws was found in CIA group than in control group. As expected, this damage could be rescued by SASP and MTX treatment (Fig. 7H, I). These results are consistent with our cellular observations in FLSs and further support the role of ferroptosis in the sulfasalazine treatment on RA.

### Sulfasalazine inhibited synovial inflammatory response in CIA mice

**Fig. 8:**
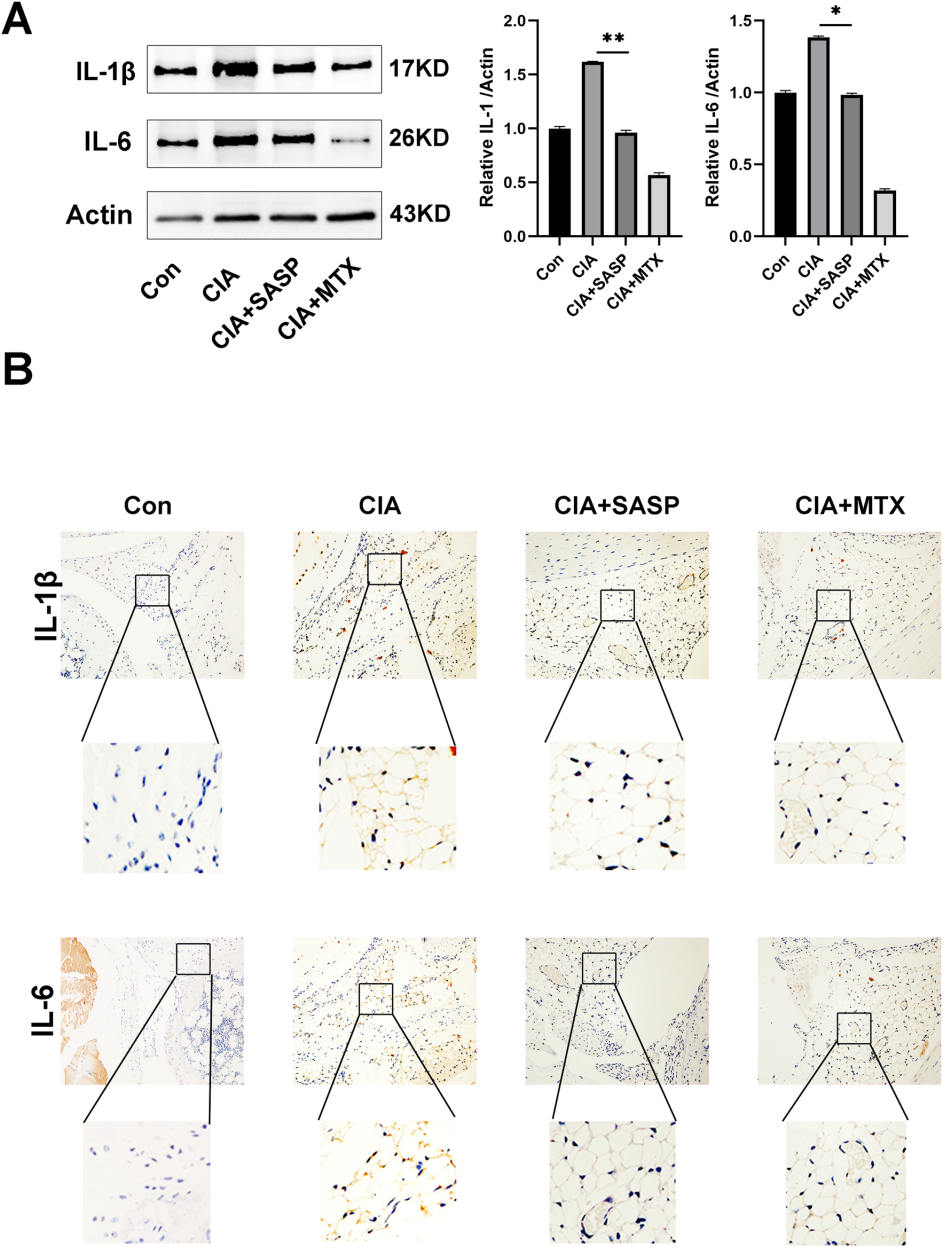
Effect of SASP on expression of inflammatory cytokines in CIA mice. DBA/1 mice were divided into four groups: control group, CIA group, CIA + SASP group and CIA + MTX group. **A** Western blot was used to detect and quantify the expression of IL-1β and IL-6 proteins in synovium of mice in the 4 groups. **B** Original magnification, up: ×100, down: ×400, immunohistochemical staining of IL-1β and IL-6. Student’s *t* test was used to analyze the experimental data (*p < 0.05, **p < 0.01, ***p < 0.001, ^#^*p* < 0.0001)

We next examined the effect of sulfasalazine on the inflammatory response in CIA mouse model. Western blot results showed that the levels of IL-1β and IL-6 in the CIA group were higher than those in the control group. After SASP and MTX treatment, the elevated expression of these inflammatory cytokines in the CIA group was blocked (Fig. 8A). Immunohistochemistry data also showed a similar trend (Fig. 8B).

### Sulfasalazine induced ferroptosis in the synovium of CIA mice

**Fig. 9:**
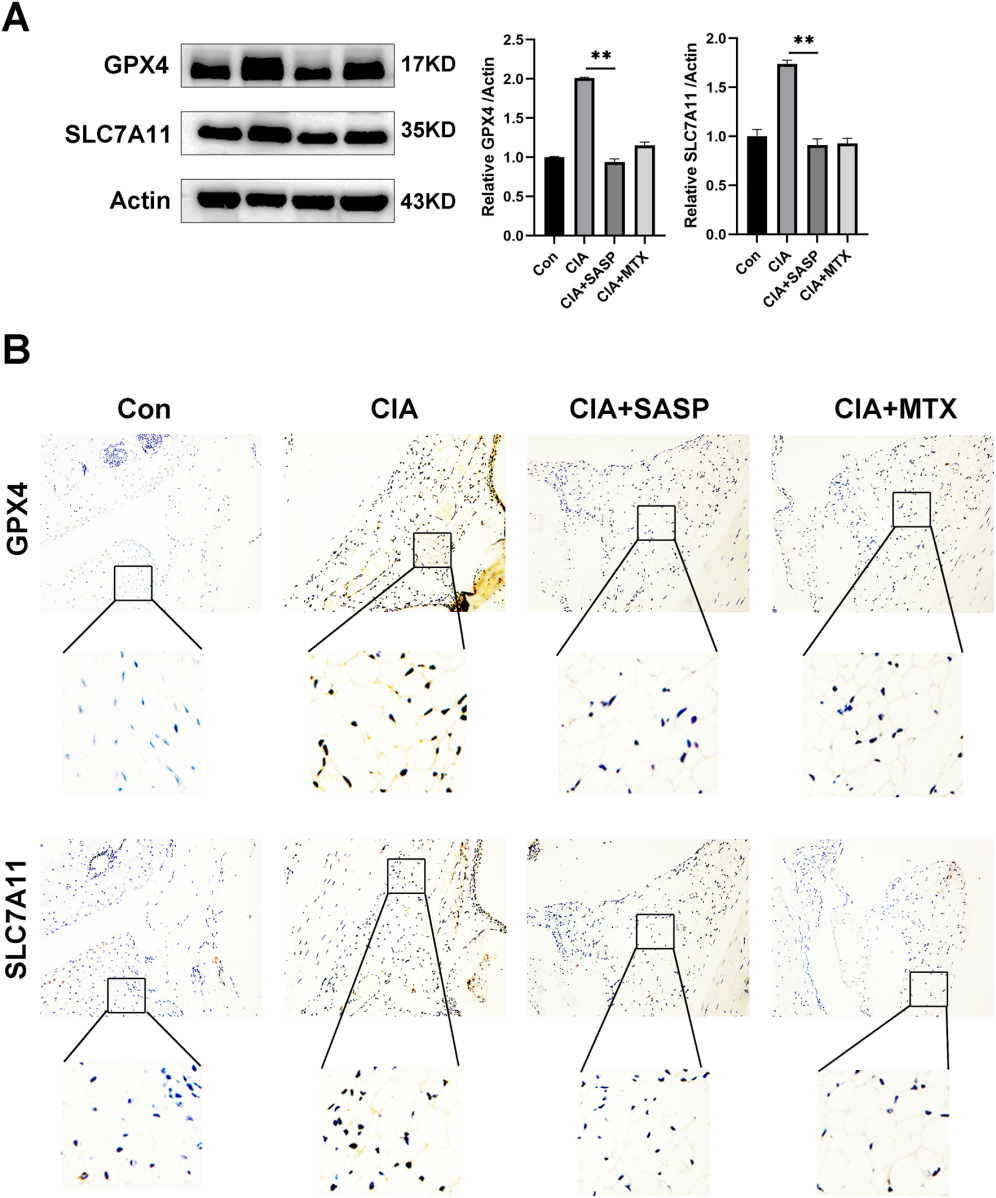
Effect of SASP on pathological changes and expression of related proteins in CIA mice after ferroptosis. DBA/1 mice were divided into four groups: control group, CIA group, CIA + SASP group and CIA + MTX group. **A** Western blot was used to detect and quantify the expression of GPX4 and SLC7A11 in the synovium of mice in the 4 groups. **B** Original magnification, up: ×100, down: ×400, GPX4 and SLC7A11 immunohistochemical staining. Student’s *t* test was used to analyze the experimental data (*p < 0.05, **p < 0.01, ***p < 0.001, ^#^*p* < 0.0001)

Finally, we examined the ferroptosis induced by sulfasalazine in synovial of CIA mice. Western blot data showed that the expressions of GPX4 and SLC7A11 in CIA group were higher than those in control group while the SASP and MTX treatment inhibited the expressions of GPX4 and SLC7A11 (Fig. 9A). The results of immunohistochemistry analysis also exhibit the similar expression pattern, which indicates that sulfasalazine could promote ferroptosis in vivo (Fig. 9B).

## Discussion

The synovium is the inner layer of the joint capsule, which is reddish, smooth, thin, soft, and composed of loose connective tissue (Luo et al. 2022). All structures in the human articular cavity, except articular cartilage and meniscus cartilage, are wrapped by a synovial membrane, which secretes synovial fluid and plays an influential role in joint activity (Qiu et al. 2014). There are two types of synovial cells: A and B. Type A macrophage-like cells have filopodia on the surface and phagocytic function while type B fibroblast-like synoviocytes play an important role in the destruction of RA joints (Bergstrom et al. 2019; Bi et al. 2019; Mariano et al. 2020; Miao et al. 2021). In this study, we demonstrated that sulfasalazine could inhibit the proliferation, migration and invasion of FLSs. The inflammatory response was reduced in FLSs by sulfasalazine treatment. At the same time, we found that sulfasalazine could reduce the expression of ferroptosis-related proteins GPX4, FTH1, FTL through regulating the PI3K-AKT-ERK1/2 and P53 pathways. In animal model experiments, intragastric administration of sulfasalazine alleviated synovial inflammation and joint swelling in CIA mice. Our findings suggest that sulfasalazine treats RA by promoting ferroptosis in FLSs.

Recent studies have shown that the migration and invasion of FLSs play a crucial role in cartilage destruction. Once FLSs reach cartilage, they can activate osteoclasts to erode and destroy bone and cartilage (Bartok and Firestein 2010; Zou et al. 2017; Cai et al. 2021). As demonstrated by scratch assay and transwell assay, sulfasalazine significantly inhibited the proliferation, migration, and invasion of FLSs. Sulfasalazine, a conventional drug widely used to treat inflammatory diseases, was also used in the treatment of RA, but the mechanism is still unclear (Zhuang et al. 2021). Xie et al. found that ferroptosis can be induced by substances in certain normal cells such as erastin and RSL3, or clinical drugs such as sulfasalazine, sorafenib, and artesunate (Xie et al. 2016). Meanwhile, Yang et al. found that AUR, an anti-rheumatoid arthritis drug, could induce ferroptosis. Therefore, we used sulfasalazine to treat RA by promoting ferroptosis in FLSs (Yang et al. 2020).

One of the main features of ferroptosis is ROS accumulation, which can be achieved in two ways: the production of iron-dependent ROS through the Fenton reaction and NADPH-dependent lipid peroxidation and GSH depletion (Yang et al. 2014). In this study, we found that the expression of FTH1 and FTL in RA-FLSs were decreased after sulfasalazine treatment, indicating the decrease of intracellular iron storage and the increase of iron uptake. In BODIPY-C11 assay, the degree of ROS accumulation also increased with increasing sulfasalazine concentration. Xie et al. found that GSH depletion leads to GPX4 reduction. In our study, we found that the expression of GPX4 in FLSs decreased after sulfasalazine treatment. Interestingly, treatment with the iron chelator DFO reversed all of these effects to some extent. This was consistent with our expectation that sulfasalazine could promote ferroptosis in FLSs. In the CIA model, through H&E staining and molybdenum targeting, we found that joint damage was less severe in CIA mice in the sulfasalazine-treated group than in CIA mice in the control group, indicating the protective role of sulfasalazine in the physiological function of the joint structure. Through immunohistochemical experiments, we found that GPX4 expression was decreased in the synovium of CIA mice treated with sulfasalazine, indicating that GPX4 inhibit synovial proliferation and slowed RA progression by suppressing ferroptosis.

Chen et al. found that galangin could inhibit ferroptosis by activating the PI3K-Akt pathway. Galangin treatment significantly rescued the phosphorylation levels of PI3K and AKT, and the anti-ferroptosis of galangin could be counteracted by the PI3K inhibitor LY294002 (Chen et al. 2022). Meanwhile, Lu et al. found that ropivacaine could inhibit the PI3K-AKT pathway to promote ferroptosis in ovarian cancer cells (Lu et al. 2022). Liu et al. found that the reduction of NRF2 induced by CdTe QDs leads to the ERK1/2 phosphorylation, which activated ferritinophagy and caused the degradation of FTH1 in lysosomes and proteasomes. As a result, the level of released free iron ions increased, which triggered ferroptosis (Liu et al. 2022). In this study, we found that the PI3K-AKT-ERK pathway is related to synovial cell ferroptosis. With the increase of sulfasalazine concentration, p-PI3K, p-AKT, and p-ERK gradually decreased. PI3K-AKT activator 1,3-dicaffeolquinic and ERK activator LM22B-10 rescued the expression levels of the all the above phosphorylated proteins. The cell viability was also increased compared with sulfasalazine group. Jiang et al. found that P53-mediated cell cycle arrest senescence and apoptosis are key barriers to cancer development, p53 suppresses cystine uptake and promotes ferroptosis by inhibiting the expression of SLC7A11 (Jiang et al. 2015). Meanwhile, Liu et al. found that activation of p53 alone was not sufficient to directly induce ferroptosis, but p53 was able to modulate the ferroptosis response in the presence of ferroptosis inducers such as GPX4 inhibitors or high levels of ROS (Liu et al. 2020). In this study, we found P53 inhibitor pifithrin-α inhibited P53 expression, but the cell viability was higher than that of SASP treatment group. By comparison, the P53 inhibitor P-α treatment result higher level recovery of cell viability than PI3K-AKT-ERK1/2 activator. Thus, sulfasalazine mainly plays its role through the p53-SLC7A11 pathway rather than the PI3K-AKT-ERK1/2 pathway.

In summary, our study suggested that sulfasalazine could induce FLSs ferroptosis through the PI3K-AKT-ERK1/2 and P53-SLC7A11 pathway, but the P53 pathway plays a more critical role, which provides new insight for the pathological and translational study of RA.

## Data Availability

The datasets used in the present study are available from the corresponding authors upon reasonable request.
